# Leukocyte esterase and alpha-defensin in periprosthetic joint infection: predictive quality and correlation in a prospective study

**DOI:** 10.1007/s00264-023-05914-7

**Published:** 2023-08-16

**Authors:** Leonard Grünwald, Florian Schmidutz, Philipp Döttger, Felix Erne, Anna Janine Schreiner, Philipp Hemmann

**Affiliations:** 1 Department of Traumatology and Reconstructive Surgery, BG Unfallklinik Tuebingen, Eberhard Karls University Tuebingen, Schnarrenbergstrasse 95, 72076 Tuebingen, Germany; 2 Department of Orthopaedic Surgery, Physical Medicine and Rehabilitation, University of Munich (LMU), Munich, Germany; 3 Orthozentrum Rosenheim, Äußere Münchener Str. 94, 83026 Rosenheim, Germany; 4 Siegfried Weller Institute for Trauma Research, BG Unfallklinik Tuebingen, Eberhard Karls University Tuebingen, Tuebingen, 72076 Germany

**Keywords:** PJI, LE, Leukocyte esterase, Periprosthetic joint infection, Alpha-defensin, Total joint arthroplasty

## Abstract

**Purpose:**

Periprosthetic joint infection (PJI) is a rare but serious complication of total joint arthroplasty (TJA). An accurate diagnosis of PJI preoperatively does not exist. Alpha-defensin (AD) is a proven and common indicator. The diagnostic marker of leukocyte esterase (LE) promises some advantages: feasibility, availability, and fast result reporting. The aim of this study was the evaluation of the predictive quality and correlation between both diagnostic tools in the diagnosis of PJI.

**Methods:**

A prospective study was conducted between April 2018 and August 2022. All patients with suspicion of PJI on hip and knee joint were included and underwent a routine and standardized joint punction. For laboratory diagnostics of AD, the synovial liquid was analyzed by ELISA. The sample was additionally applied to a LE test strip (Combur 10 Test, Roche Diagnostics, Mannheim, Germany).

**Results:**

A total of 249 patients were examined (mean age 67.12 ± 11.89; gender distribution man/woman 139 (55.8%)/110(44.2%), hip/knee 71(28.5%)/178 (71.5%). According to EBJIS criteria, PJI was diagnosed in 54 (21.7%) patients. AD showed excellent results with an AUC of 0.930 (sensitivity/specificity 0.870/0.990). LE yielded very good results with an AUC of 0.820 (sensitivity/specificity 0.722/0.918). Both parameters showed a strong positive correlation.

**Conclusion:**

LE is a rapidly available alternative in PJI diagnostics. The simultaneous determination of both markers may enhance diagnostic reliability. A routine usage may shorten the time from diagnosis to treatment of PJI.

## Introduction

Total joint arthroplasty (TJA) is one of the most commonly performed surgical procedures. With the worldwide ageing of society, an increased number of joint replacements is expected [1, 2]. Periprosthetic joint infection (PJI) is a rare but serious and devasting complication and is accompanied by a high burden of disease [3].

The infection rate after hip replacement is usually less than 1% and after knee replacement less than 2% [4]. Nevertheless, due to the increasing number of joint replacements, a consecutive increase number of periprosthetic joint infections (PJI) is expected [5].

A growing number of research studies are focusing on this challenging pathology. The findings were incorporated into the recommendations of the Musculoskeletal Infection Society (MSIS), European Bone and Joint Infection Society (EBJIS), and Infectious Diseases Society of America (IDSA). The most popular research topics on PJI focus on diagnostics, antibiotics, and risk factors [6]. Nevertheless, there is still no gold standard regarding PJI diagnostics, which aggravates an early and accurate treatment.

In the current literature, several laboratory tests are available to detect PJI. However, none of them can exactly detect PJI and thus are determined as the gold standard. In any case, early diagnosis and calculated treatment are crucial factors for decision-making and treatment of infection [4].

Alpha-defensin (AD) has recently been induced and is a well-evaluated and proven test used for PJI diagnostics [7–10]. AD can be tested by commercial test kits (e.g., Synovasure™) or machine-aided by enzyme-linked immunosorbent assays (ELISA). However, the method is patent-protected, expensive, and not ubiquitously available. The use of culture media for enrichment and cultivation of suspicious bacteria from synovial fluid requires a time span of up to 14 days.

Recently, leukocyte esterase (LE) has been proposed as an additional marker for detecting PJI. The main advantages of the LE marker are as follows: readily available, cheap availability, and fast result reporting. The LE strip tests deliver the final result in less than two min with a sensitivity and specificity of 0.89, respectively 0.86 [8]. The increasing importance of LE is also reflected in various PJI definitions as a listed minor criterion [11, 12]. Both the International Consensus Meeting (ICM) definition and the Musculoskeletal Infection Society (MSIS) definition for PJI assign three points to LE, which indicates a similar diagnostic accuracy as for alpha-defensin. In the literature, there are only a few studies with small case numbers comparing LE and AD directly [13, 14].

The aim of this study was to evaluate (I) the predictive quality (sensitivity, specificity, positive and negative predictive value) of LE and AD in the diagnosis of PJI in patients with total hip arthroplasty (THA) and total knee arthroplasty (TKA) as well as (II) the correlation between LE and AD in a large cohort.

## Materials and methods

### Study cohort

Between April 2018 and August 2022, all patients with a routine joint punction due to suspicion of PJI were included prospectively in this study for LE testing of the affected joint. The following inclusion criteria were applied: TJA of the hip or knee joint and concurrent suspicion of acute or chronic PJI. This includes clinical symptoms ranging from fulminant joint sepsis with clear signs of infection to more indolent symptoms, such as pain or joint dysfunction [15]. The following exclusion criteria were provided: Native joints, age < 18 years, and tumor disease.

### Definition of PJI

European Bone and Joint Infection Society (EBJIS) criteria of 2021 were used for the confirmation of PJI [15]. This means, as a short summary:

- Purulence around the prosthesis or sinus tract
- Increased synovial fluid leukocyte count
- Positive histopathology
- Or significant microbial growth in synovial fluid, periprosthetic tissue, or sonication fluid [16]

### Joint puncture method

All patients gave informed legal consent. The procedure of joint puncture was performed under sterile conditions, with fluoroscopy used for hip aspiration The centre of the joint was entered at the relevant landmark perpendicular to the skin using a thin needle. Every joint puncture was performed by experienced physicians and assisted by medical staff.

### Data collection method

After successful aspiration, the fluid was centrifugated at 1000 rpm for 10 minprior to being tested. Then one drop of supernatant was applied to a LE test strip (Combur 10 Test, Roche Diagnostics, Mannheim, Germany). According to the four colour grades on the box (negative, ~ 10–25, ~ 75, ~ 500 leukocytes/µl), the result could be read from the color patch change on the LE strip after 2 min. Furthermore, the aspirates were tested for synovial leukocyte cell count, PMN (%), and CRP in our laboratory. Alpha-Defensin samples were sent on the same day to a collaborating laboratory (Labor Dr. Fenner and colleagues, Hamburg, Germany) where a standard enzyme-linked immunosorbent assay (ELISA) was performed. In addition, joint aspirates were applied to blood culture mediums (aerobic and anaerobic) and a microbiological swab tube with following incubation for 14 days.

### Approval by the ethics committee

This study was approved by the local ethics committee (189/2018BO2).

### Statistical analysis

For statistical analyses, IBM SPSS Statistics for Windows, version 23.0 (IBM Corp., Armonk, NY, USA), was used. Chi-squared test (for categorical variables) and univariate analysis of variance were used to test the null hypothesis. The level of significance was set at *p* ≤ 0.05. Receiver operating characteristics (ROC) were calculated to analyze the diagnostic performance of alpha-defensin and LE. The area under the curve (AUC) and 95% confidence interval (CI) were calculated. According to the EBJIS criteria for PJI diagnosis sensitivity, specificity, positive predictive value (PPV), and negative predictive value (NPV) were calculated for LE and alpha-defensin. Spearman’s rank correlation *ρ* was used to determine the relationship between variables of interest.

## Results

Between March 2018 and August 2022, a total sample of 249 patients was examined. The mean age was 67.12 ± 11.89 years (range 24 to 91, with 55.8% men (*n* = 139) and 44.2% women (*n* = 110). A PJI according to the EBJIS criteria was diagnosed in 54 (21.7%) patients. The subjects’ descriptive data are summarized in Table 1. There were no significant differences between the PJI cohort and the non-PJI group.

**Table 1:** Treated side, age, localization, and gender in accordance with PJI diagnosis

	Sum	Side		Age	Localization		Gender	
PJI	N (%)	Left	Right	M (SD)	Hip	Knee	Male	Female
Yes	54 (21.7%)	33 (24.8%)	21 (18.1%)	67.8 (12.84)	13 (18.3%)	41 (23.0%)	36 (25.9%)	18 (16.4%)
No	195 (78.3%)	100 (75.2%)	95 (81.9%)	66.9 (11.65)	58 (81.7%)	137 (77.0%)	103 (74.1%)	92 (83.6%)
Total	249 (100%)	133 (53.4%)	116 (56.6%)	67.1 (11.89)	71 (28.5%)	178 (71.5%)	139 (55.8%)	110 (44.2%)

The data for synovial AD, leukocytes, PMN, and CRP is summarized in Table 2. All variables showed significant differences between the two groups.

**Table 2:** Mean and standard deviation for AD, CRP, leukocytes, nucleated cells, and polymorphonuclear according to PJI and corresponding statistics for univariate analysis of variance

	PJI		Statistics
	Yes	No	
AD numerical	2.86 (± 1.86)	0.07 (± 0.20)	F(1) = 425.358; p ≤ .001*
CRP mg/l	2.12 (± 3.59)	0.23 (± 0.43)	F(1) = 39.956; p ≤ .001*
Leukocytes 1/µl	30,206.57 (± 43,247.79)	934.49 (± 1350.16)	F(1) = 87.654; p ≤ .001*
Polymorpho-nuclear cells (%)	75.59 (± 21.32) Range: 13–98	29.94 (± 19.82) Range: 2–87	F(1) = 215.626; p ≤ .001*

Spearman-rank correlations were conducted to detect connections between the diagnosis of PJI and variables of interest (Table 3).

**Table 3:** Correlation of LE, AD, CRP, leukocytes, polymorphonuclear, and microbiology with PJI

	Correlations (*r*) with PJI (yes/no)
LE *N* = 249	*r* = .679** *p* ≤ .001
AD *N* = 249	*r* = 892** *p* ≤ .001
CRP *N* = 188	*r* = .424** *p* ≤ .001*
Leukocytes *N* = 244	*r* = .672** *p* ≤ .001
Polymorphonuclear *N* = 244	*r* = .609** *p* ≤ .001
Microbiology (incubation 24 h) *N* = 246	r = .395** *p* ≤ .001
Microbiology (incubations 48 h) *N* = 247	*r* = .484** *p* ≤ .001

**Fig. 1:**
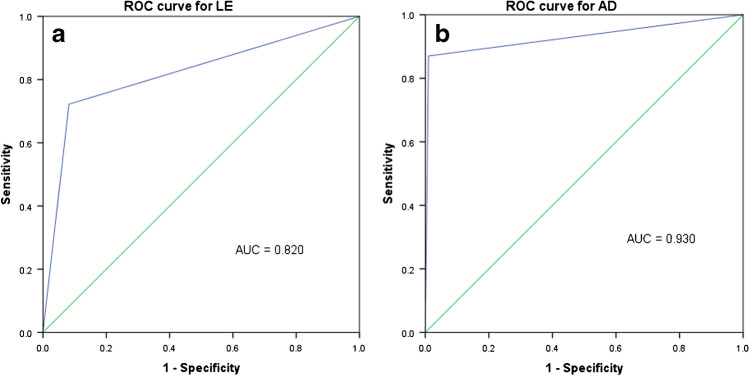
**a** Receiver operating characteristics (ROC) curve for LE (0.820); **b** receiver operating characteristics (ROC) curve for AD (AUC: 0.930)

The ROC analysis for LE showed an AUC of 0.820 for LE (Fig. 1), which indicates that it is a reliable test to determine PJI (Fig. 1). Sensitivity for LE was 0.722 and specificity 0.918 with a threshold of 10–25 leukocytes/µl. For the AD, the AUC is 0.930. Therefore, the ROC analysis indicated that AD is a very excellent test for detecting PJI (Fig. 1). The sensitivity for AD was 0.870, and specificity was 0.990 when the threshold was ≥ 1. The Spearman-rank correlations for LE and alpha-defensin were *r* = 0.696 (*p* ≤ 0.001*) indicating a strong correlation.

Diagnostic accuracy for LE showed a sensitivity (specificity) of 0.722 (0.918) and for AD 0.870 (0.990) (Table 4).

**Table 4:** Diagnostic accuracy for LE and AD respectively for the diagnosis of PJI. CI, confidence interval; TP, true positive; FP, false positive; FN, false negative; TN, true negative

Test	AUC (95% CI)	Best threshold	Sensitivity	Specificity	PPV	NPV	TP–FP–FN–TN
LE	0.820 (0.746 to 0.895)	10–25 leukocytes /µl	0.722	0.918	0.709	0.922	39–16–15–179
AD	0.930 (0.877 to 0.984)	≥ 1	0.870	0.990	0.959	0.965	47–2–7–193

## Discussion

### Predictive quality of AD in the diagnosis of PJI

This prospective study evaluated the predictive quality of LE and AD in the diagnosis of PJI in patients with THA and TKA. In line with the current literature, AD showed excellent results with an AUC of 0.930. Yu et al. showed an AUC of 0.875 in their study with 65 infected patients and 65 non-infected patients [13]. In our study, the sensitivity of 0.870 and specificity of 0.990 were better than in the study of Yu et al. with 0.831 and 0.862 [13]. Sharma et al. examined AD in 107 patients using the Synovasure™ test and demonstrated an AUC of 0.916, and they concluded that AD is an “outstanding” biomarker for PJI [17]. In a current study by Kuo et al., AD showed an AUC of 0.92 and was listed as the best diagnostic tool in the minor criteria from the 2018 International Consensus Meeting (ICM) [18]. A similar result could be shown by Levent et al. in a study with 260 patients [19]. AD showed an AUC of 0.922 which was classified as an outstanding preoperative minor criterion. The meta-analysis by Tang et al. pointed out that synovial AD is one of the best independent preoperative diagnostic tests with an AUC of 0.98 [20]. It reported a sensitivity of 0.91 and specificity of 0.96 of AD in their meta-analysis [20]. Overall, the results for AD in this study are consistent with current literature. The actual finding supports the importance and value of AD in the diagnosis of PJI.

### Predictive quality of LE in the diagnosis of PJI

Similarly, in this study, LE yielded very good results with an AUC of 0.820. Yu et al. showed in their study with 130 patients an AUC of 0.854 if considered 500 leukocytes as threshold before and after centrifugation [13]. If 250 and 500 leukocytes were considered threshold after centrifugation, Yu et. al. could even enhance the AUC to 0.877. Furthermore, sensitivity (specificity) enhanced as well from 0.754 (0.954) to 0.800 (0.954). In the present study, sensitivity (specificity) was slightly lower with 0.722 (0.918) which might be related to a smaller cohort of PJI patients. [13]. Li et al. reported about the influence of centrifugation when interpreting LE strip tests [21]. They reported that the colour change after centrifugation resulted in a lower grade and therefore recommended a lower threshold when centrifugation was used. Since all samples in this study were centrifuged, it could be assumed that the best threshold is 10–25 leukocytes/µl. It should be mentioned that the risk of false positive results is high which is shown in Table 4 with 16 false-positive results. Additional obstacles could result from the variances of the test kits. Sharma et al. used two different test kits for LE and reported different results for sensitivity (specificity) with 90% and 81% (84% and 95%) [17]. Levent et al. used the same test kit as in this study and examined 260 patients and found a sensitivity (specificity) of 78% (91.4%) [19]. With 109 patients, the PJI cohort was nearly twice as big compared to this study which might explain the better diagnostic accuracy of LE compared to this study.

### Direct comparison between AD and LE

Direct comparison between AD and LE was only examined in a few studies with small cohorts. Deirmengian et al. compared sensitivity and specificity of synovial AD and LE in 46 patients [22]. Regarding AD, they showed a sensitivity and specificity of 100%. Referring to LE, a sensitivity of 69% and a specificity of 100% were reported. [22]. The authors concluded that AD immunoassay outperformed the LE test strips. Our results show a similar tendency. The findings show that sensitivity and specificity of AD were also better than for LE. At this point, it must be clarified that De Vecchi et al. report contrary results. The authors found a better diagnostic accuracy for LE with 95.5% compared to AD with 89.4% [14]. All the above-named studies included less patients in both cohorts than our study. Yu et al. could show that LE has a similar diagnostic accuracy compared to AD [13]. Spearman correlation showed a strong positive correlation between AD and LE. However, a causal relationship may not be assumed between these two markers. The correlation underlines the diagnostic value of LE and is seen as an equivalent minor criterion in the diagnosis of PJI [13].

### Limitations of the study

There are several limitations to this study. The cohort with PJI is small which might underestimate the diagnostic accuracy of LE. Studies with larger infected cohorts showed better results for LE than the present study. Secondly, all joint aspirations were centrifugated with a possible downgrading when interpreting the LE stripes. This might also affect the diagnostic accuracy of LE. Finally, different manufacturers of the LE strips are available and provide different gradings which makes a direct comparison to other studies less informative.

### LE strips as a diagnostic tool

The major benefit of using LE strip tests is the rapid delivery of the results within two min. The procedure can be assessed “at the bedside” or “in the theatre” and requires no special training or equipment. As discussed above, the different manufacturers may have diagnostic variances regarding their strips. The use of LE strips in PJI can be assumed as a useful diagnostic tool in emergency cases, intraoperatively, or where AD is not available at all. A routine usage may shorten the time from diagnosis to treatment, compared to complex laboratory diagnostics. The simultaneous determination of several markers, especially LE and AD, may further enhance diagnostic reliability.

## Conclusion

The present study shows excellent results for AD and very good results for LE Diagnosing PJI in TJA, with both parameters showing a strong positive correlation. Therefore, LE is a cheap and rapidly available alternative in PJI diagnostics, especially if AD is not available or decision-making is necessary. Furthermore, simultaneous determination of both markers may enhance the diagnostic reliability.

## Data Availability

The dataset used and/or analysed during the current study are available from the corresponding author on reasonable request.
